# Electroacupuncture enables semaglutide dose sparing in type 2 diabetes by boosting drug concentration and suppressing β-cell apoptosis

**DOI:** 10.3389/fendo.2026.1880819

**Published:** 2026-08-26

**Authors:** Xinyi Yang, Jiayue Wu, Bing Gao, Yunmao Zhu, Yuchen Sun, Ling Ouyang, Bufan Wu, Wenxiao Lan, Jing Zhang, Yujie Zhang, Binbin Yuan, Yitong Sun, Yuanbing Zhu, Shuping Fu, Xinyue Jing, Bin Xu

**Affiliations:** 1 Key Laboratory of Acupuncture and Medicine Research of Ministry of Education, Nanjing University of Chinese Medicine, Nanjing, China; 2 Traditional Chinese Medicine Hospital of Ruichang, Jiujiang, China; 3 Nantong Hospital of Traditional Chinese Medicine, Nantong, China; 4 School of Chinese Materia Medica, Beijing University of Chinese Medicine, Beijing, China; 5 Acupuncture and Moxibustion College, Chengdu University of Traditional Chinese Medicine, Chengdu, Sichuan, China

**Keywords:** electroacupuncture, glucagon-like peptide-1 receptor, pancreatic β-cell, semaglutide, type 2 diabetes mellitus

## Abstract

**Purpose:**

Semaglutide is effective for type 2 diabetes mellitus (T2DM) but limited by dose-dependent gastrointestinal adverse effects. As electroacupuncture (EA) possesses glucose-lowering effects, this study investigates the therapeutic potential and mechanism of combining EA with semaglutide to facilitate dose reduction while maintaining efficacy.

**Methods:**

Male *db/db* mice were divided into: the model group(T2DM), the high/normal/low-dose semaglutide group (SH, SN, SL), the normal/low-dose semaglutide + EA at ST25 group (ST25N, ST25L), and the EA at ST25 group (ST25). The *db/m* mice served as the control group (CON). Blood glucose regulation was assessed via random/fasting blood glucose and oral glucose tolerance test (OGTT). To evaluate glycemic control and lipid profiles, HbA1c and insulin levels were quantified via ELISA, and T-CHO, TG, LDL-C, and HDL-C via biochemical kits. GLP-1R expression and β-cell abundance were assessed by immunofluorescence, and pancreatic apoptosis by TUNEL staining. PKA, pPKA, Bcl-2, Bax, and GLP-1R were analyzed by Western blot. Male Sprague–Dawley (SD) rats were separated into 2 groups: the normal-dose semaglutide rats group (RSN) and the normal-dose semaglutide + EA at ST25 rats group (RST25N). LC-MS/MS was employed to measure the concentrations of semaglutide in the plasma and pancreas, thereby evaluating the influence of EA.

**Results:**

EA enhances the effects of semaglutide on improving glycemic control and lipid metabolism, with significant reductions in random/fasting blood glucose, HbA1c, TCHO, TG and LDL-C levels, improvements in OGTT, insulin and HDL-C levels, and longer duration of controlled blood glucose within the target range, Furthermore, EA combined with semaglutide showed a marked increase in pancreatic β-cells, with the increased expression of pancreatic GLP-1R, PKA, pPKA and Bcl-2, the reduced expression of Bax, increased concentration of semaglutide in plasma and pancreas.

**Conclusion:**

EA combined with normal- or low-dose semaglutide achieved glycemic control comparable to, or even better than, high-dose semaglutide alone, and its mechanism may be related to its enhancement of GLP-1R to inhibit β-cell apoptosis and the increased concentration of semaglutide in plasma and pancreas. EA thus has great potential to reduce the dose of semaglutide to alleviate its side effects while ensuring its clinical efficacy.

## 1 Introduction

Diabetes mellitus is a chronic disease with a high incidence worldwide with type 2 diabetes mellitus(T2DM) accounting for over 90% of cases and contributing significantly to morbidity and mortality worldwide (1). T2DM arises from insulin resistance and progressive β-cell dysfunction, often necessitating lifelong medication (2, 3). While conventional therapies like insulin supplementation can effectively regulate blood glucose, they are frequently associated with undesirable effects, including weight gain and hypoglycemia, and fail to halt disease progression (4, 5). Thus, strategies that address both metabolic control and reducing side effects are urgently needed.

Semaglutide, a glucagon-like peptide-1 receptor agonist (GLP-1RA), potentiates glucose-dependent insulin secretion, suppresses glucagon release, delays gastric emptying, and promotes satiety (6). These effects not only improve glycemic control but also facilitate weight reduction, which is particularly beneficial for overweight or obese T2DM patients (7–12). It mimics native glucagon-like peptide-1 (GLP-1) function by stimulating insulin secretion and suppressing glucagon release in a glucose concentration-dependent manner, thereby effectively lowering blood glucose levels (13, 14). However, recent reports of its dose-dependent gastrointestinal side effects and other side effects have highlighted that it is necessary to seek an auxiliary intervention that can reduce dosage while maintaining or enhancing its efficacy (15, 16).

The clinical status and role of acupuncture in the management of diabetes mellitus are steadily advancing. In April 2021, acupuncture was for the first time incorporated into Chapter 19 of the *Chinese Clinical Guidelines for the Prevention and Treatment of Type 2 Diabetes (2020 Edition)*. The guideline explicitly recognizes the role of acupuncture in the prevention and management of type 2 diabetes and recommends its use in combination with conventional therapy to improve glycemic control (17). Electroacupuncture (EA) has also gained interest for its ability to modulate blood glucose through neural, endocrine, and immunological pathways (18, 19). Many studies have demonstrated that EA applied to specific acupoints (e.g. ST25, Tianshu) reduces blood glucose (20, 21). Furthermore, our previous work has demonstrated the synergistic potential of EA as an adjunctive therapy across various diseases (22–24). Specific examples include EA enhancing the function of metformin (22) and, in a separate study, combining with an HDAC1 inhibitor to suppress tumor growth (25). These results point to a promising role for EA as an adjuvant therapy by augmenting the therapeutic efficacy of drugs. Recent studies have further shown that EA can regulate the GLP-1R/PKA signaling pathway and improve glucose metabolism in diabetic models (26). Given that semaglutide exerts its therapeutic effects via GLP-1R, a molecular target shared with EA, we hypothesized that combining these two interventions would provide complementary therapeutic benefits in T2DM.

Despite established efficacy of both EA and semaglutide in managing T2DM, the therapeutic potential of their combination remains largely unexplored. To address these gaps, this study was designed to investigate the synergistic effect of EA at ST25 combined with semaglutide in *db/db* mice. Based on previous evidence demonstrating that EA improves glucose metabolism and enhances the therapeutic efficacy of drugs, we hypothesized that EA will enhance the function of semaglutide, thereby enabling a reduction in the required drug dosage while maintaining optimal therapeutic outcomes. Such a strategy would be clinically valuable because lowering semaglutide dosage may reduce dose-dependent gastrointestinal adverse events while maintaining glycemic control.

## 2 Materials and methods

### 2.1 Animals

70 male *db/db* mice and 10 male *db/m* mice aged 6–8 weeks were purchased from GemPharmatech Company (Production License No.: SCXK (Zhe) 2019-0002). Fifty male Sprague–Dawley (SD) rats aged 6–8 weeks were purchased from Shanghai Bikai Keyi Biotechnology Co., Ltd (Production License No.: SCXK (Hu) 2023-0009). The mice and rats were housed in the SPF-level animal facility at the Experimental Animal Center of Nanjing University of Chinese Medicine, under a 12 h light/dark cycle, with *ad libitum* access to food and water, ambient noise ≤ 60 dB, barrier temperature of 22 ± 2 °C, and humidity of 55 ± 10%. These experiments were approved by the Animal Ethics Committee of Nanjing University of Chinese Medicine (Animal license:202310A073, 202509A060, 202509A061).

### 2.2 Electroacupuncture and pharmacological treatment

#### 2.2.1 Part1

This study aims to determine the impact of different interventions on glycemic control in *db/db* mice and investigate the cellular and molecular mechanisms responsible for the resulting alterations.

##### 2.2.1.1 Experiment 1

After one week of adaptive feeding, 10 male *db/m* mice were assigned to the control group (CON). 70 *db/db* mice with fasting blood glucose (FBG) levels above 11.1 mmol/L were allocated into seven groups according to their FBG levels and glucose tolerance test results: the model group(T2DM), the high-dose semaglutide group (SH), the normal-dose semaglutide group (SN), the low-dose semaglutide group (SL), the normal-dose semaglutide + EA at ST25 group (ST25N), the low-dose semaglutide + EA at ST25 group (ST25L), and the EA at ST25 group (ST25) (27). All animals were maintained on a high-fat diet (HFD), containing 31% lard for the duration of the experiment.

**Figure 1:**
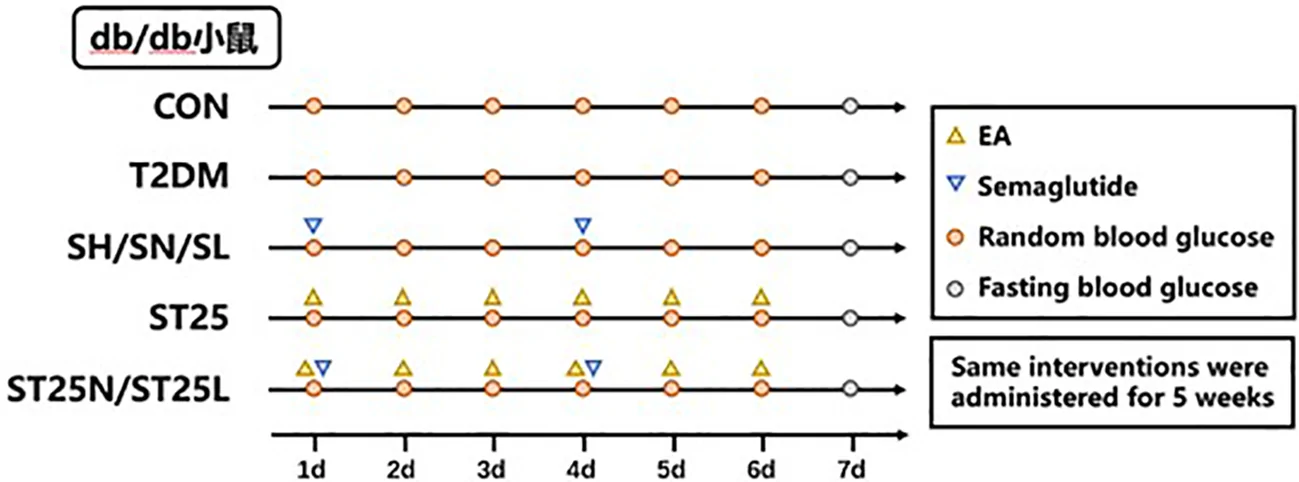
Experimental grouping and interventions in *db/db* and *db/m* mice. *db/m* mice were assigned to the CON group. *db/db* mice were divided into T2DM, SH, SN, SL, ST25, ST25N, ST25L groups to evaluate the therapeutic effect of EA combined with semaglutide.

The SH, SN and SL groups received subcutaneous injections of semaglutide at doses of 0.13 mg/kg, 0.065 mg/kg and 0.03125mg/kg, respectively, twice weekly for five weeks, while the Con and T2DM groups were administered an equal volume of saline on the same schedule. The ST25N and ST25L group received combined therapy, consisting of subcutaneous semaglutide (0.065 mg/kg and 0.03125 mg/kg) and EA stimulation. During EA sessions, mice were anesthetized using inhalational isoflurane anesthesia. For EA treatment, a pair of acupuncture needles (Beijing Zhongyan Taihe Medical Instrument Co., Ltd., China) were perpendicularly inserted unilaterally into the ST25 acupoint (located 5 mm lateral to the intersection point of the upper 2/3 and lower 1/3 of the line between the xiphoid process and the upper margin of the pubic symphysis) at a depth of 3–5 mm (28). The needles were connected to a Han’s electroacupuncture instrument (Nanjing Jisheng Medical Technology, Nanjing, China), and stimulation parameters were set at 2 mA and 2/15 Hz for 20 minutes per session. Stimulation was given every day, 6 times a week, as a course of treatment. It is operated by skilled and licensed acupuncturists throughout the five weeks. The ST25 group received EA alone following the same protocol. To control for anesthesia effects, all groups that did not undergo EA received 20 minutes of anesthesia matching the duration of EA sessions (**Figure 1**).

#### 2.2.2 Part2

This study aimed to quantify and compare the plasma and pancreas concentrations of semaglutide in rats following different treatments, thereby clarifying the potential mechanism underlying the significantly enhanced hypoglycemic effect observed with the combined EA and semaglutide intervention.

##### 2.2.2.1 Experiment 2

After adaptive feeding, SD rats were randomly divided into two groups using the randomization function in SPSS software: the normal-dose semaglutide rats group (RSN) and the normal-dose semaglutide + EA at ST25 rats group (RST25N), with 5 rats in each group on the first day, the RST25N group received subcutaneous injections of semaglutide (0.045 mg/kg) and underwent EA stimulation at the ST25 acupoint immediately after administration. For the subsequent 6 days, EA stimulation was repeated daily at the same time without drug administration. Rats were anesthetized with inhalational isoflurane during the EA procedure. EA was applied for 20 minutes with parameters setting at 2 mA and 2/15 Hz, The RSN group were administered the same dose of semaglutide (0.045 mg/kg) and were anesthetized for 20 minutes consistent with the RST25N group. Blood samples were collected​ at 5, 30 min, 1, 2, 4, 6, 8, 12, 24, 36, 48, 72, 96, 120 and 144 h after semaglutide administration.

##### 2.2.2.2 Experiment 3

After adaptive feeding, 40 SD rats were randomly divided into two groups: the normal-dose semaglutide rats group (RSN) and the normal-dose semaglutide + EA at ST25 rats group (RST25N), with 20 rats in each group. The interventions for the RSN and RST25N groups were consistent with those in Experiment 2, except that the semaglutide dosage was adjusted to 0.36 mg/kg. Pancreas samples were collected at 4, 8, 12, 24 h after administration.

### 2.3 Measurement of random and fasting blood glucose levels

The FBG was measured weekly after 12 h fasting (with free access to water) using a blood glucose meter (Roche, USA). Random blood glucose (RBG) was measured six times per week at a fixed time each day using a blood glucose meter (**Figure 1**).

### 2.4 Oral glucose tolerance test (OGTT)

The OGTT was conducted before and after the whole treatment. After 12 h fasting, mice were orally administered 2.0 g/kg glucose. Blood glucose was measured at 0,30,60,90 and 120 min after administration using a blood glucose meter (29).

### 2.5 Enzyme-linked immunosorbent assay (ELISA)

Serum levels of insulin and HbA1c were quantified using ELISA kits (Jiangsu Meibiao Biological Technology Co., Ltd, China). The procedures were performed following instructions of ELISA kits.

### 2.6 Biochemical analysis

The lipid profile in serum, including triglycerides (TG), total cholesterol (T-CHO), low-density lipoprotein cholesterol (LDL-C), and high-density lipoprotein cholesterol (HDL-C), was assessed with corresponding assay kits (Jiancheng Bioengineering Institute, Nanjing, China). All procedures were performed in accordance with the manufacturers’ protocols.

### 2.7 Bioanalysis of semaglutide in plasma and pancreas

The rats were sacrificed 4,8,12,24 hours after interventions. Pancreas was quickly removed on ice. The pancreatic tissue was weighed, homogenized with methanol. An aliquot of 950 μL of homogenate was taken, and 50 μL of internal standard (IS) liraglutide was added. After vortexing for 5 min, the mixture was centrifuged at 14,000 rpm for 15 min at 4 °C. Calibration curves for concentration of semaglutide in pancreas was 0.0019–0.5 ng/mL. The supernatant was separated and evaporated under a stream of nitrogen (CentriVap Concentrator, Labconco, Germany). Calibration curves for concentration of semaglutide in plasma were 0.006–12.5 ng/mL. For the plasma samples, 100 μL of rat plasma was combined with 50 μL of the IS solution and 250 μL of methanol. The samples were vortexed for 10 min and subsequently centrifuged at 15,000 × g for 10 min. Calibration curves for concentration of semaglutide in pancreas was 0.006-12.5 ng/mL (30).

The concentrations of semaglutide plasma and pancreas were quantified using a Triple Quad 6500+ system consisting of Exion LC (AB Sciex, Japan) and Triple Quad 6500+ (AB Sciex, Singapore). Chromatographic conditions: Acquity™ UPLC BEH C18 Column (130 Å, 1.7 μm, 2.1×100 mm; Waters Acquity, Ireland), mobile phase A (0.1% formic acid in water; Thermo Fisher, China) and B (acetonitrile; Merck Millipore, Germany) with gradient elution as **Table 1**, column temperature 40 °C, flow rate 0.3 mL/min, injection volume 2 μL. Mass spectrometry conditions: ESI source, MRM mode, spray voltage 5.5 kV, nebulizer temperature 350 °C, gas 1 (55 mL/min), curtain gas (45 mL/min), gas 2 (55 mL/min). Transitions: 1029.3→1238.2 (semaglutide) and 938.8→1128.7 (liraglutide). Data were processed using Sciex OS 2.1.6 Analytics.

**Table 1:** Gradient conditions for semaglutide in rats.

Time (min)	0.1% FA in DW (%)	ACN (%)	Flow rate (mL/min)
0	70	30	0.3
1.0	70	30	0.3
8.0	5	95	0.3
9.0	5	95	0.3
9.5	70	30	0.3
10.0	70	30	0.3

ACN, acetonitrile; DW, distilled water; FA, formic acid.

### 2.8 Immunofluorescence staining

Immunohistochemistry was performed on pancreas sections, blocked with 3% BSA, incubated with glucagon-like peptide-1 receptor (GLP-1R) antibody, glucagon antibody, insulin antibody, TUNEL kits (servicebio, Wuhan, China, G1502) at 4 °C overnight, followed by secondary antibodies (servicebio, Wuhan, China) (1:300) and DAPI (servicebio, Wuhan, China, G1012). Images were captured using a Thunder Imager Model Organism (Leica Microsystems Srl, Buccinasco, Italy). A list of all primary antibodies was provided in **Supplementary Table 1**.

### 2.9 Western blot (WB)

Total protein was extracted with RIPA lysis buffer (protease inhibitors added), quantified via BCA assay, separated by SDS-PAGE, and transferred to PVDF membranes. After blocking, membranes were incubated with GLP-1R, protein kinase A (PKA), phosphorylated PKA (pPKA), B-cell lymphoma -2 (Bcl-2), Bcl-2 associated X protein (Bax) and Tubulin, β-actin antibodies overnight, followed by HRP-labeled secondary antibodies (1:2000) (CST, USA). Bands were visualized using ECL, and gray values were analyzed with Image J. The relative expressions of PKA, pPKA, Bcl-2 and Bax were normalized to Tubulin, and GLP-1R expression was normalized to Actin. Detailed information for all primary antibodies was provided in **Supplementary Table 1**.

### 2.10 Statistical analysis

Data are expressed as mean ± SEM. IBM SPSS Statistics 27.0.1 was used for one-way ANOVA with LSD and Tamhane’s T2 for *post-hoc* comparisons and Two-way ANOVA was used with Bonferroni for *post-hoc* comparisons. *P* < 0.05 was considered statistically significant. Data analysis was performed by investigators blinded to group allocation.

## 3 Results

### 3.1 EA potentiates the glycemic control of semaglutide in *db/db* Mice

**Figure 2:**
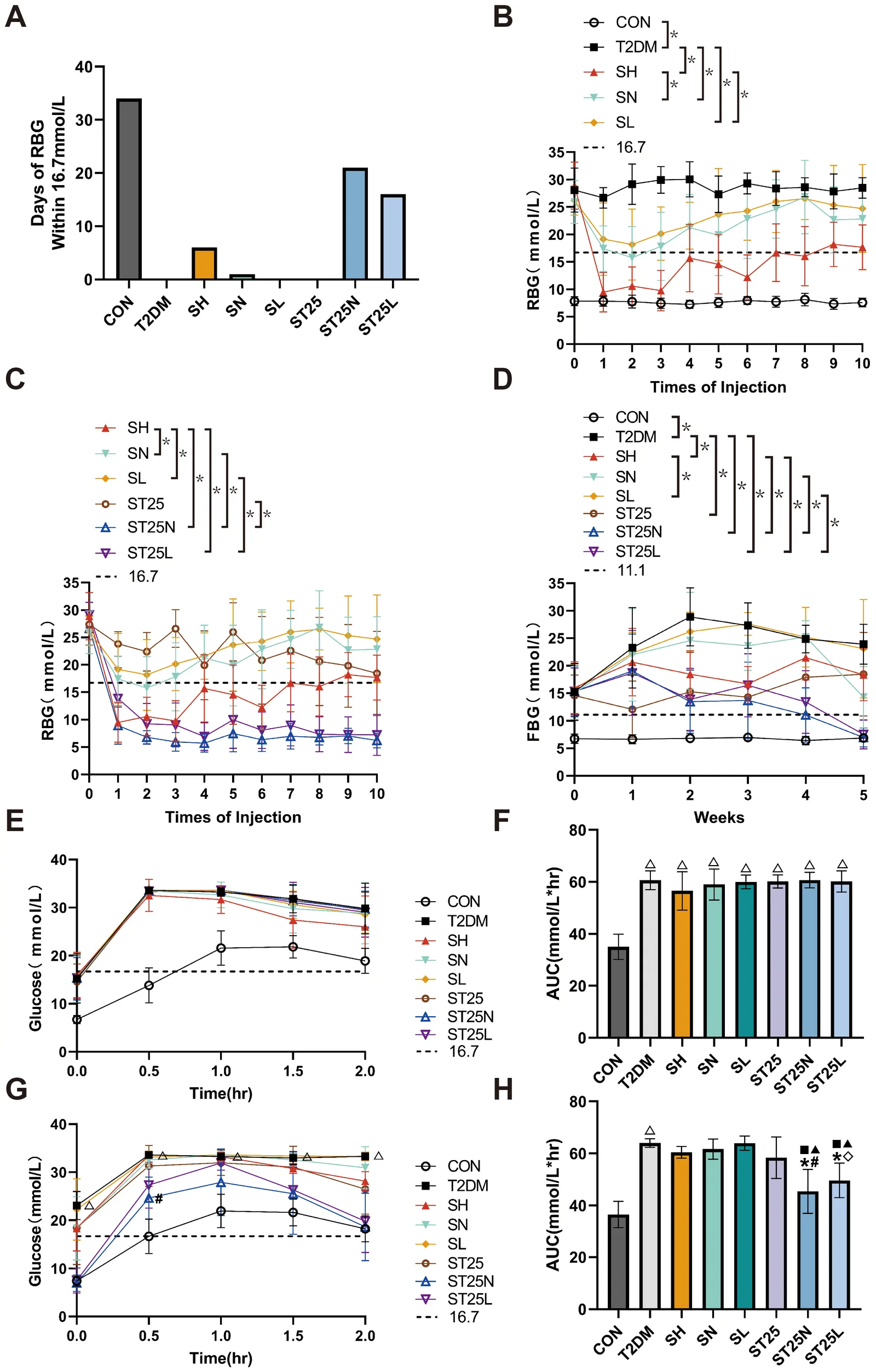
The combination of EA and semaglutide enhances glycemic control in *db/db* mice. **(A)** The number of days that the RBG of each group remained within 16.7mmol/L. **(B, C)** 24-hour Random blood glucose levels after each injection. (n=7-10) **(D)** Fasting blood glucose levels of each group within 5 weeks. (n=7-10) Data are presented as mean ± SEM. Two-way ANOVA was used for **(B-D)** with Bonferroni for *post-hoc* comparisons, *P < 0.05. **(E)** Initial oral glucose tolerance test (OGTT) and **(F)** corresponding area under the curve (AUC); (n=10) **(G)** final OGTT after 5 weeks intervention and **(H)** corresponding AUC. (n=7-10) ^△^*P* < 0.05 vs. CON group; *P < 0.05 vs. T2DM group; ^▪^*P* < 0.05 vs. SH group; ^#^*P* < 0.05 vs. SN group; ^⋄^*P* < 0.05 vs SL group; ^▴^*P* < 0.05 vs ST25 group.

Although EA and semaglutide effectively control glycemia individually, it remains unknown whether their combined use offers additive or synergistic benefits. The *db/db* mice’s blood glucose level was significantly higher than that of the control group (*P* < 0.05) at the beginning as shown in (**Supplementary Figures 1A, B**; **Supplementary Table 2**). During the intervention period, the hyperglycemia of the T2DM group was maintained, but the RBG levels in all the treatment groups were dose-dependently lowering from the first treatment (**Supplementary Figures 1A–C**; **Supplementary Table 2**). Surprisingly, the hypoglycemic effects in EA combined with normal or low dose of semaglutide (ST25N or ST25L) groups were even stronger than high-dose semaglutide (SH) group (*P* < 0.01) (**Supplementary Figure 1C**; **Supplementary Table 2**). Meanwhile, the ST25N and ST25L groups exhibited more days with blood glucose within the glycemic target range (< 16.7 mmol/L), which was defined as the target because this threshold is widely used to indicate effective glycemic control in diabetic rodent models rather than normalization of blood glucose levels (31), compared to the SH, SN and SL groups during the treatment and this effect was also dose-dependent (**Figure 2A**; **Supplementary Figure 1C**; **Supplementary Table 2**, **3**). We also found that semaglutide’s glucose-lowering effect in SH, SN and SL groups gradually diminished over time. It was difficult to lower blood glucose to normal levels from the second injection in SN and SL groups. Even in SH group, the blood glucose can’t be controlled within 16.7mmol/L from the 7th injection (**Figure 2B**; **Supplementary Figure 1B**; **Supplementary Table 2**, **3**) In contrast, the ST25N and ST25L groups consistently achieved glycemic targets for two days after each administration and showed more stable glucose control throughout compared with the SH, SN and SL groups (**Figure 1C**; **Supplementary Figure 1C**; **Supplementary Table 2**, **3**). It also revealed that treated with EA alone can decrease the blood glucose gradually. However, during the treatment process, the blood sugar levels were all not within 16.7mmol/L. The blood glucose level in EA group was comparable to the SH group after the last treatment (**Figure 2C**; **Supplementary Figure 1C**; **Supplementary Table 2**, **3**).

Taking a close look at the 24-hour RBG levels after each administration, the SH group showed a significant reduction in 24-hour glucose levels compared with the T2DM group, but the glucose levels increased gradually after the fourth injection. However, the ST25N and ST25L groups consistently maintained blood glucose within the target range, even equivalent to the CON group and exhibited a more pronounced hypoglycemic effect than the other intervention groups (**Figures 2B, C**; **Supplementary Table 3**). These results suggest that the combination of EA and normal-dose/low-dose semaglutide can achieve or even exceed the hypoglycemic efficacy of high dose semaglutide alone.

FBG levels of the *db/db* mice were significantly higher than that in the control mice (**Figure 2D**; **Supplementary Table 4**). During treatment, the FBG levels decreased in a dose-dependent manner in both the SN group and the SH group, the FBG levels in the ST25N and ST25L groups remained markedly reduced compared to T2DM and other intervention groups. During the treatment, FBG levels in the two groups were lower than SN group from the second week and even lower than SH group from the 4th week. At the last week, the FBG levels only in the ST25N and ST25L groups dropped below 11.1mmol/L (**Figure 1D**). As for the EA group, FBG levels also decreased from the first week, but increased gradually from the 3th week and was equivalent to SH and SN groups at the last week.

FBG or RBG levels could not be used as a comprehensive measure of glycemic control. Oral glucose tolerance test (OGTT) was performed before the treatment as well as the last week of the experiment to show the curative effect of each administration. Additionally, the area under the curve (AUC) for the OGTT test was computed as a measure of total glucose exposure. Glucose tolerance in *db/db* mice was deteriorative obviously (*P* < 0.05) before treatment (**Figures 1E, F**). After five weeks’ therapy, the ST25N and ST25L groups exhibited the most rapid glucose clearance, with the AUC values being substantially lower than those of the ST25, SH, SN, SL and T2DM groups (*P* < 0.05) (**Figures 1G, H**). However, in this study, glucose tolerance was not improved in each dose of the semaglutide groups, or in the EA alone group. The blood glucose level in the ST25N and ST25L groups decreased from 1h to 2h and was even decreased to control level at 2h. These results suggest that combining EA with a normal or low-dose of semaglutide can achieve glycemic control comparable to, even exceeding high dose semaglutide alone.

### 3.2 EA improves the effect of semaglutide on metabolic disorders in *db/db* mice

**Figure 3:**
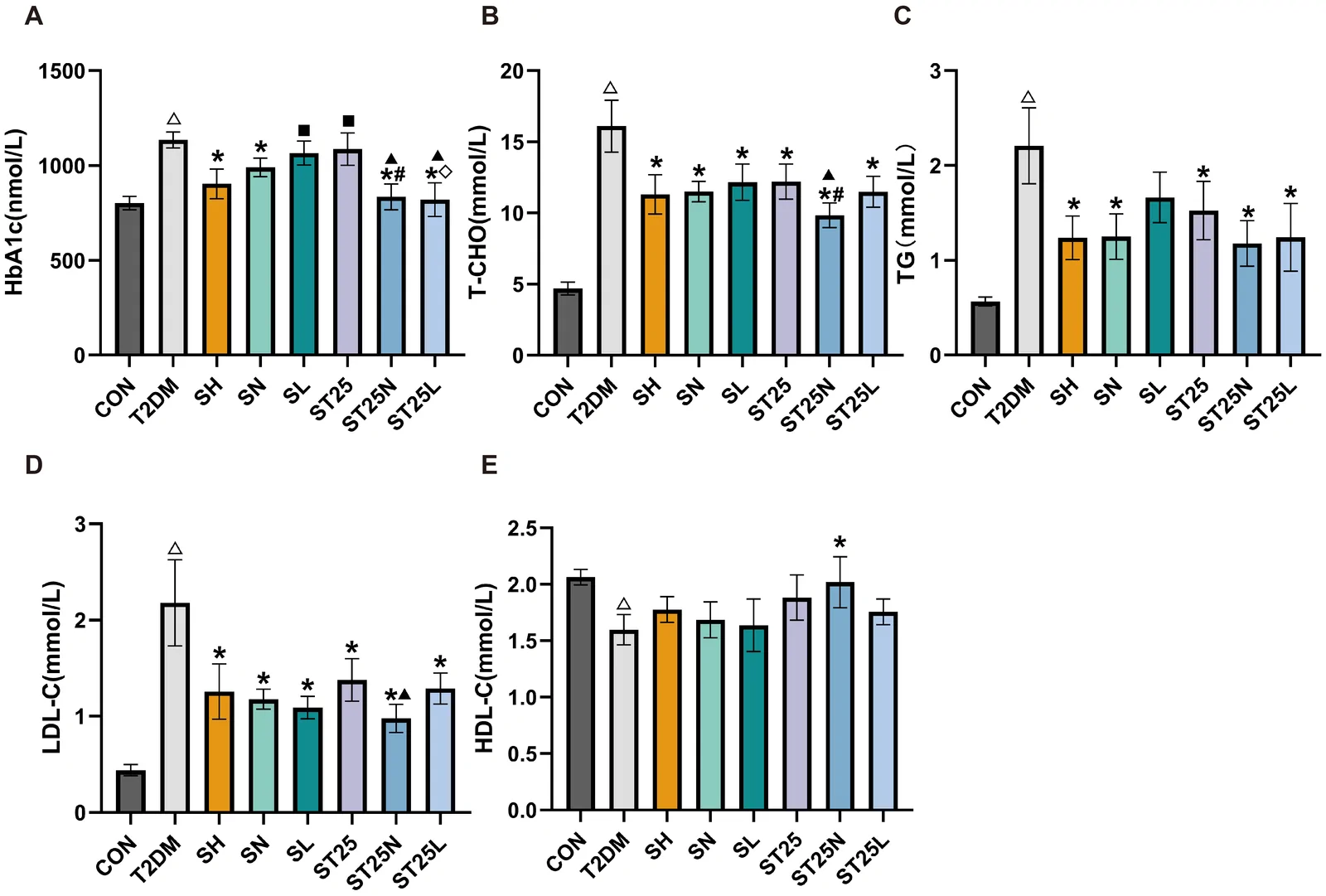
EA promotes the effects of semaglutide on glycemic and lipid metabolism in *db/db* mice. **(A)** HbA1c, **(B)** T-CHO, **(C)** TG, **(D)** LDL-C, and **(E)** HDL-C levels after 5 weeks of intervention. (n=6) Data are presented as mean ± SEM. *^△^P* < 0.05 vs. CON group; ^*^P < 0.05 vs. T2DM group; ^▪^*P* < 0.05 vs. SH group; *^#^P* < 0.05 vs. SN group; ^⋄^*P* < 0.05 vs SL group; *^▴^P* < 0.05 vs ST25 group.

To evaluate the protective effects of combined EA and semaglutide on metabolic disorders in mice, Hemoglobin A1c (HbA1c) and lipid metabolism were assessed. HbA1c has remained the standard biomarker for glycemic control (32). The HbA1c levels of the *db/db* mice were higher than those in the control mice (*P* < 0.05, **Figure 3A**). After five weeks of intervention, HbA1c significantly decreased in SH, SN, ST25N and ST25L group. The decrease of HbA1c in SH, SN groups was dose-depended. The ST25N group showed a greater reduction in HbA1c compared to the SN group, and ST25L also decreased greatly compared to the SL group (*P* < 0.05, **Figure 3A**), which suggests that semaglutide combined with EA provides a better glycemic control compared to using semaglutide alone at an equivalent dose. In terms of lipid metabolism, dyslipidemia was found in *db/db* mice. All intervention groups showed significant reductions in T-CHO (*P* < 0.01) and LDL-C (*P* < 0.01) compared with the T2DM group, and SH, SN, ST25, ST25N and ST25L decreased significantly compared with the T2DM group in TG (*P* < 0.01), suggesting both EA and semaglutide play positive roles in modulating lipid metabolism in mice. Notably, the ST25N group exhibited a marked reduction in T-CHO (*P* < 0.01) compared to both the SN and ST25 groups. Concomitantly, only the ST25N group demonstrated a significantly higher level of HDL-C (*P* < 0.01) relative to the T2DM group (**Figures 3B–E**), which highlight the benefit of combining use of EA and semaglutide. These evidences strongly suggest that the combined therapy is more effective than either using semaglutide or EA alone in correcting metabolic disorders.

### 3.3 EA enhances the concentration of semaglutide in plasma and pancreas after subcutaneous injection to rats

**Figure 4:**
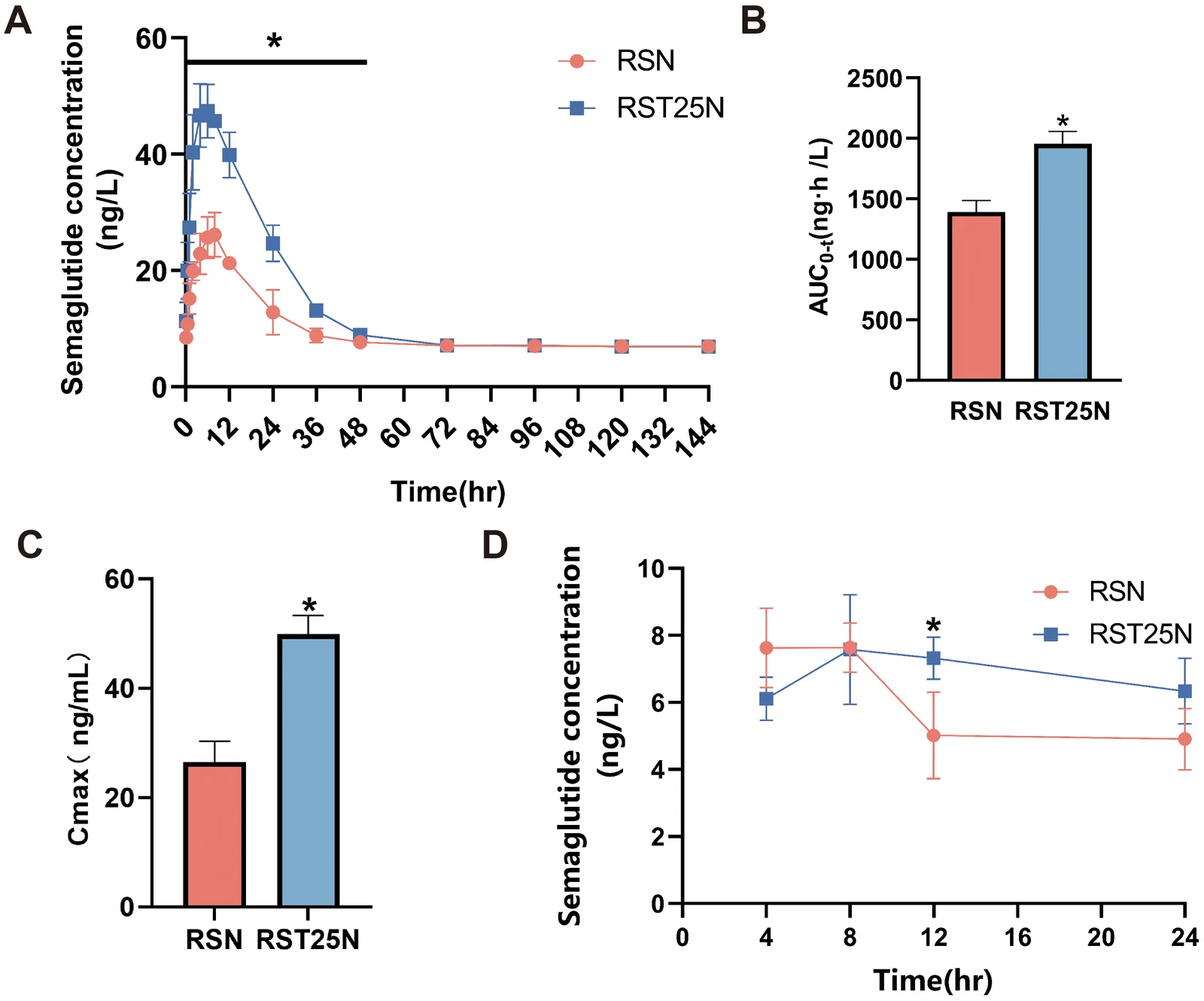
EA enhances the systemic and pancreatic concentration of semaglutide in rats. **(A)** Semaglutide concentration in plasma. (n=5) **(B)** AUC and **(C)** Cmax of semaglutide in plasma. (n=5) **(D)** Semaglutide concentration in pancreas. (n=5) Data are presented as mean ± SEM. ^*^P < 0.05 vs. RSN group.

Given that the combination of EA and semaglutide elicits superior blood glucose lowering and metabolic improvements, it is crucial to investigate whether EA alters the pharmacokinetic profile of semaglutide, particularly its concentration in the plasma and pancreas. We found that EA treatment increased the plasma concentration of semaglutide, characterized by an increased AUC and maximum concentration (Cmax), significant difference was found in AUC values (*P* < 0.01, **Figure 4A**) and Cmax (*P* < 0.01, **Figures 4B, C**) compared with the non-EA group. This effect was also markedly observed in pancreas. Although the RSN group exhibited a higher concentration of semaglutide at the 4-hour time point, the RST25N group demonstrated consistently higher tissue concentrations at the subsequent time points, with a significant difference at 12 hours (*P* < 0.01, **Figure 4D**). The increased concentrations of semaglutide in the plasma and pancreas provide a plausible explanation for the enhancement in blood glucose control, suggesting that improved drug concentration may attribute to the synergistic effect of EA.

### 3.4 EA promotes the pancreatic β-Cell protective effect of semaglutide

**Figure 5:**
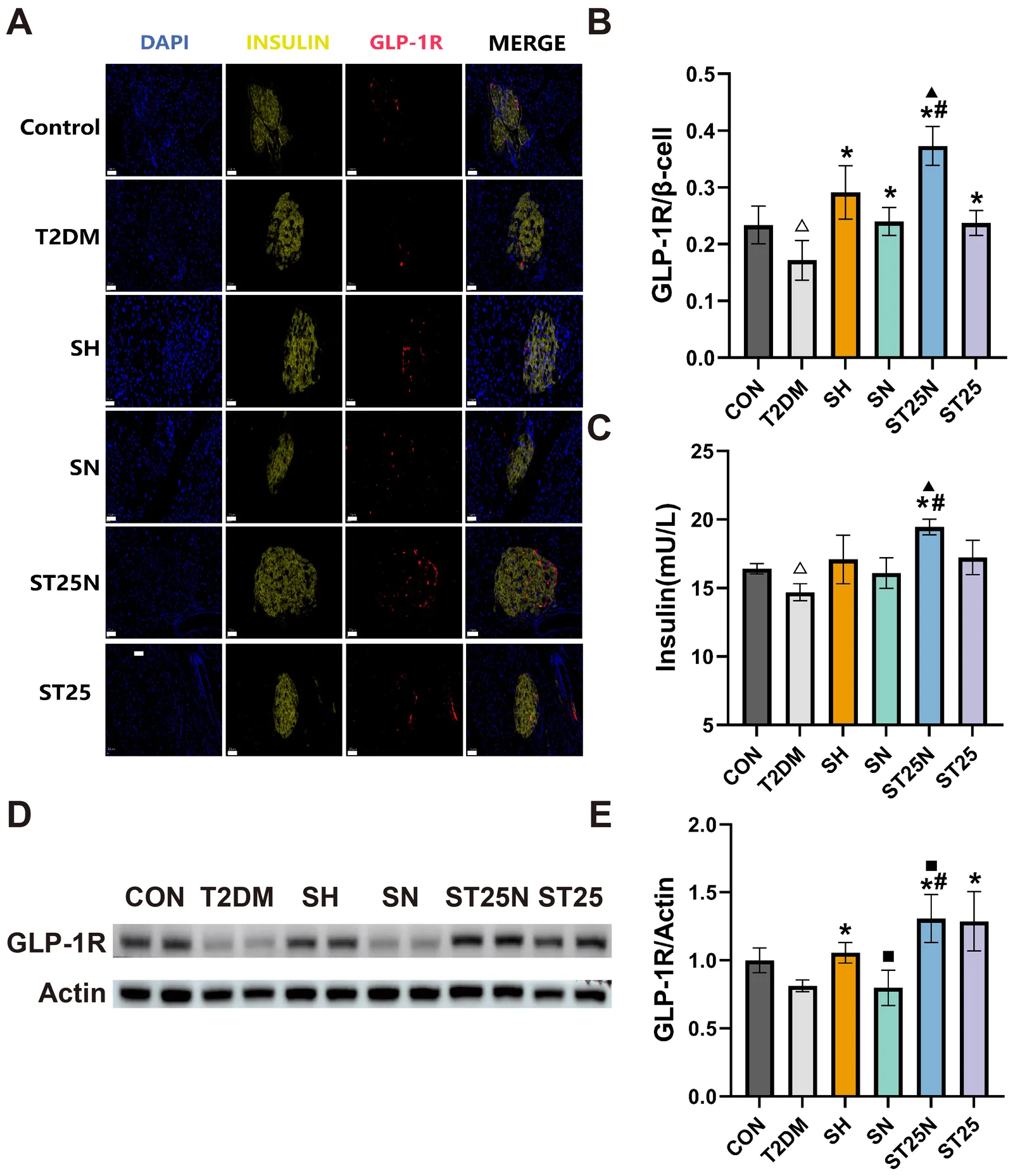
Combined EA and semaglutide treatment upregulates GLP-1R expression and enhances insulin secretion in pancreatic β-cells. **(A)** Representative immunofluorescence images of pancreatic β-cell and GLP-1R (400x) and **(B)** relative expression of GLP-1R in β-cell. **(C)** Insulin levels measured in serum. (n=5) **(D)** Representative western blot images of GLP-1R. **(E)** Quantification of GLP-1R protein levels. (n=3) *^△^P* < 0.05 vs. CON group; ^*^P < 0.05 vs. T2DM group; *^▪^P* < 0.05 vs. SH group; *^#^P* < 0.05 vs. SN group; *^▴^P* < 0.05 vs ST25 group.

To quantify the changes in pancreatic GLP-1R expression following the combined therapy, we performed immunofluorescence staining. The results showed a marked upregulation of GLP-1R expression in pancreatic β-cells in all intervention groups compared to the T2DM group (*P* < 0.05). Notably, the ST25N group showing a more pronounced increase compared to the SN group (*P* < 0.05, **Figures 5A, B**). Meanwhile, corresponding insulin levels in serum were significantly elevated in the ST25N group relative to the SN, ST25 and T2DM groups (*P* < 0.05, **Figure 5C**). Subsequently, Western blot analysis consistently revealed a significant increase in pancreatic GLP-1R expression in the SH, ST25N, and ST25 groups (*P* < 0.05, **Figures 5D, E**). Notably, GLP-1R expression in the ST25N was even higher than SN and ST25 groups. Previous studies have shown that GLP-1R has protective effects on pancreatic β-cells (33–35). Therefore, we can assume that the combination of EA and semaglutide may provide superior protection for pancreatic β-cells, and further suggest that this benefit may be achieved through the activation of the GLP-1R signaling pathway.

### 3.5 EA synergistically inhibits pancreatic β-Cell apoptosis with semaglutide

**Figure 6:**
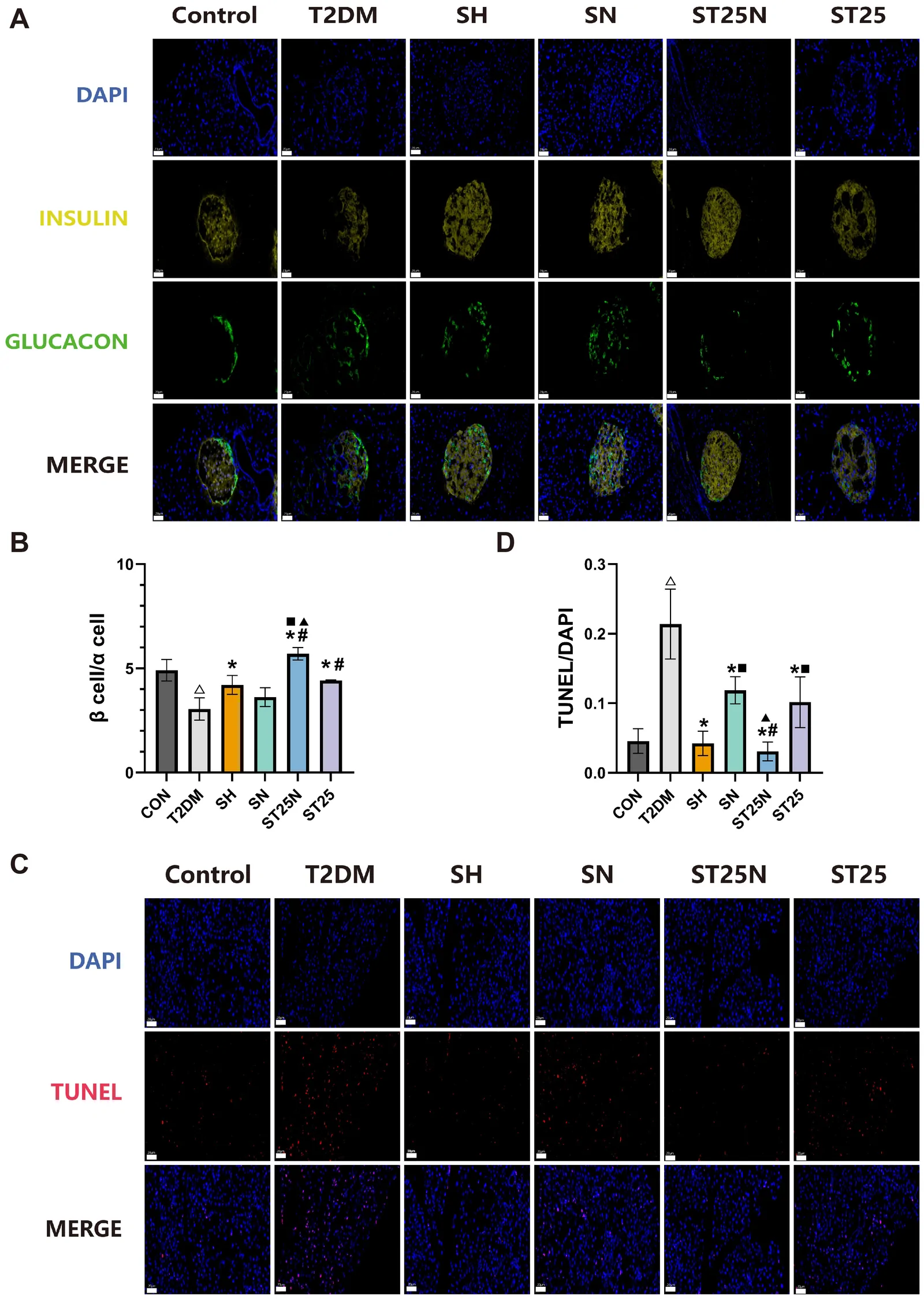
Combined EA and semaglutide treatment enhances survival and suppresses apoptosis of β-cell. **(A)** Representative immunofluorescence images of pancreatic islets stained for β-cell (insulin) and α-cell (glucagon) (400x) and **(B)** relative expression of β-cell. **(C)** Representative immunofluorescence images of TUNEL staining in pancreas (400x) and **(D)** relative expression of TUNEL cells. (n=3) Data are presented as mean ± SEM. *^△^P* < 0.05 vs. CON group; ^*^P < 0.05 vs. T2DM group; *^▪^P* < 0.05 vs. SH group; *^#^P* < 0.05 vs. SN group; *^▴^P* < 0.05 vs ST25 group.

**Figure 7:**
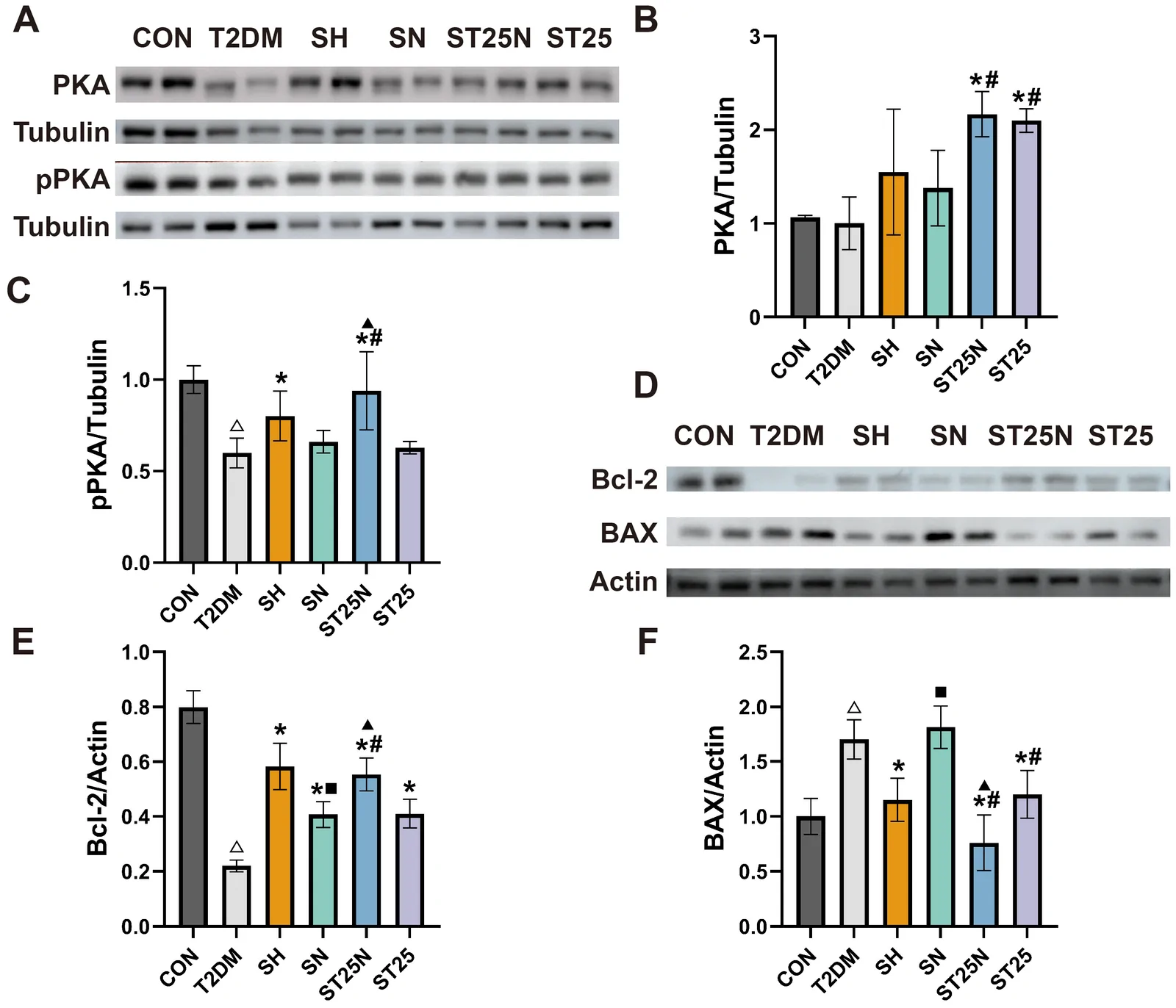
Combined EA and semaglutide treatment promotes β-cell survival and suppresses apoptosis through PKA signaling. **(A)** Representative western blot images of PKA and pPKA in pancreas. **(B, C)** Quantification of PKA and pPKA protein levels. (n=3) **(D)** Representative western blot images of Bcl-2 and Bax in pancreas. **(E, F)** Quantification of Bcl-2 and Bax protein levels. (n=3) Data are presented as mean ± SEM. *^△^P* < 0.05 vs. CON group; ^*^P < 0.05 vs. T2DM group; *^▪^P* < 0.05 vs. SH group; *^#^P* < 0.05 vs. SN group; *^▴^P* < 0.05 vs ST25 group.

We first performed immunofluorescence for both pancreatic β-cells and pancreatic α-cells to investigate the changes of the relative β-cell abundance in the different treatment groups. Comparing to T2DM group, the result demonstrated a significant increase in the relative abundance of β-cells in SH, ST25N, ST25 groups (*P* < 0.05). Meanwhile, the ST25N group showed a more notably increase compared to the SH, SN, and ST25 groups (*P* < 0.05), suggesting that the combination of EA and semaglutide may provide superior protection for β-cell survival (**Figures 6A, B**). Consistent with this finding, TUNEL staining demonstrated a significant reduction in pancreatic cell apoptosis in both SH and ST25N group compared to SN, ST25 and T2DM groups (*P* < 0.05), with ST25N group exhibiting much lower pancreatic cell apoptosis than SN and ST25 groups (**Figures 6C, D**). Hence, we further explored on β-cell apoptotic pathway. Initially, the results of WB revealed a significant increase in pPKA expression in ST25N and SH groups relative to T2DM group, with ST25N group exhibiting higher pPKA levels than SN and ST25 groups. Additionally, PKA expression was markedly upregulated in ST25N and ST25 groups compared with both T2DM and SN groups. (*P* < 0.05, **Figures 7A–C**), suggesting an activation of the PKA signaling pathway with the combined therapy. Furthermore, in terms of anti-apoptosis, Bcl-2 decreased markedly in T2DM group. All treatment groups could increase Bcl-2. Notably, ST25N group exhibited a more pronounced increase in Bcl-2 than SN and ST25 groups, even equivalent to SH group. Conversely, the expression of Bax in pancreas was markedly upregulated in T2DM group, but downregulated in the SH, ST25N, and ST25 groups respectively. Bax in ST25N was significantly lower than other treatment groups. (*P* < 0.05, **Figures 7D–F**) These results indicate that the combined treatment of EA and semaglutide effectively inhibits apoptosis of pancreatic β-cells. These results further support our hypothesis that the combination of EA and normal-dose semaglutide achieves β-cell protective effects comparable to those of high-dose semaglutide alone.

## 4 Discussion

As a long-acting GLP-1RA approved in China, semaglutide offers a potent therapeutic option for hyperglycemia. However, its use is associated with certain limitations. The most common side reactions are gastrointestinal, including nausea, vomiting, diarrhea, abdominal pain, and constipation. These symptoms are generally mild to moderate and may appear in the early treatment stage, but they tend to worsen with long-term use and increased dosages. These side effects raise concerns among both clinicians and patients, shrinking the pool of individuals who can successfully utilize this therapy (15, 16, 36). Therefore, exploring strategies to reduce the dosage while maintaining its therapeutic efficacy holds significant clinical value. We turned our attention to EA, which has demonstrated hypoglycemic effects in previous studies. Earlier research also suggests that EA can exert a synergistic effect with metformin in lowering blood glucose (22). However, in our experiment, EA alone was unable to lower blood glucose to the normal range, which is consistent with earlier reports indicating that hardly can EA reduce blood glucose below 16.7 mmol/L (37, 38). The combination of EA and semaglutide significantly improved HbA1c levels, and the significant reduction in RBG and FBG levels in the ST25N and ST25L groups, coupled with the improvement in oral glucose tolerance and the reduction in blood glucose AUC, indicating that the combined intervention more effectively enhanced glucose metabolism stability than semaglutide or EA alone. Furthermore, the dose administered to mice in our study was calculated based on the dose used in human. However, the corresponding dose cannot maintain a week-long hypoglycemic effect as it does in humans. Consequently, we adopted a plan of dosing twice per week (39). After the first injection, the SH group-maintained blood glucose within the target range for two days, but this duration dropped to one day thereafter. Notably, after the sixth injection, the blood glucose in this group entirely failed to reach the target range. In contrast, the ST25N group consistently sustained blood glucose within the target range for two days since the first injection, and extended to three days after the ninth injection. The ST25L group also exhibited a progressive improvement during the interventions period. As the number of administrations increased, the number of days in which blood glucose control reached target increased from 1 to 3 days. The total number of days that blood glucose reached target in the ST25N and ST25L groups was significantly higher than that of any single drug treatment group, including the SH group. This shows that electroacupuncture combined with semaglutide can not only increase its short-term hypoglycemic effect, but also significantly improve its stability and sustainability of blood glucose control.

In addition to improving blood glucose homeostasis, semaglutide and EA also have significant regulatory effects on lipid metabolism (15, 40). Our results demonstrate that the combined therapy produced superior amelioration of metabolic dysfunction, as evidenced by a more substantial improvement in lipid profile compared to monotherapies. These findings demonstrate that integrating EA with semaglutide therapy significantly enhances the regulation of glucose homeostasis and lipid metabolism compared to using semaglutide alone. Therefore, EA emerges as a highly promising adjuvant therapy, offering a novel clinical strategy to optimize diabetes management and improve patient outcomes. Although these findings support the therapeutic potential of EA as an adjunct to semaglutide, the present study was conducted exclusively in a diabetic mouse model. Therefore, the long-term safety and clinical applicability of this dose-sparing strategy require further validation in future studies.

Semaglutide exerts its therapeutic effects primarily by binding to GLP-1R on pancreatic β-cells. It is well-established that pharmacokinetic parameters are key determinants of the pharmacodynamic response of GLP-1RAs. Studies show that semaglutide has a clear dose-exposure-response relationship, with higher systemic exposure directly related to enhanced therapeutic efficacy, including superior glycemic control and HbA1c reduction (41, 42). Based on our previous study that EA significantly increases the brain concentration of Astragaloside IV (43), we hypothesized that EA might similarly affect the concentration of semaglutide in both plasma and the pancreas. Our results revealed that the RST25N group exhibited significantly increased AUC and Cmax values of semaglutide in plasma. Meanwhile, EA improved the concentration of semaglutide in pancreas, particularly at 12-hour after dosage. The immunofluorescence and western blot results also showed that EA combined with semaglutide enhanced pancreatic GLP-1R expression, particularly in pancreatic β-cells. The enhancement in glycemic control might be associated with increased semaglutide concentrations and pancreatic GLP-1R levels, which may facilitate greater activation of GLP-1R. The precise pharmacokinetic mechanisms underlying the increased semaglutide concentrations, however, remain to be elucidated and warrant further investigation.

Recent studies have further emphasized that GLP-1R activation protects functional β-cell mass against metabolic stress-induced injury, thereby preserving long-term insulin secretory capacity (44). Our immunofluorescence results also revealed that the combined treatment can further increase the relative abundance of β-cells than EA or semaglutide. Accordingly, the serum insulin level in the combined treatment group was the highest among all groups. The activation of GLP-1R signaling appears to be a central mechanism, which aligns with the known role of GLP-1RA in promoting β-cell survival and insulin levels (45–47). Since semaglutide exerts its therapeutic effects primarily by binding to GLP-1R on pancreatic β-cells, the synergy between EA and semaglutide likely stems from the activation of GLP-1R. Specifically, our findings of increased semaglutide concentration and upregulated GLP-1R expression in the pancreas corroborate this mechanism, suggesting that the combined intervention maximizes therapeutic outcomes by effectively preserving and potentiating β-cell function. To validate this mechanism, future studies employing pharmacological inhibition or genetic manipulation will be necessary to establish a direct causal relationship.

Previous researches have proved that activation of the GLP-1R pathway promotes cAMP generation and downstream PKA activation, which plays a central role in β-cell survival and insulin secretion (48, 49). The enhanced PKA and pPKA expressions in ST25N group indicate potentiation of this signaling pathway. Moreover, GLP-1RAs enhance the expression of the anti-apoptotic protein Bcl-2 while suppressing the activity of pro-apoptotic protein Bax to exert anti-apoptotic functions (47, 50, 51). The upregulation of Bcl-2 helps maintain mitochondrial membrane integrity, and inhibition of Bax expression further blocks the activation of the mitochondrial apoptotic pathway (52), thereby inhibiting β-cell apoptosis induced by various factors, including high glucose levels. In line with these findings, our results demonstrate that the combination of EA and normal-dose semaglutide more effectively enhances the expression of PKA, pPKA, and Bcl-2 while reducing Bax expression compared with either using EA or semaglutide alone, which leads to a greater suppression in β-cell apoptosis. This was further confirmed by TUNEL staining and the increased serum insulin level. Hence, these findings indicate that EA may serve as a complementary therapy to semaglutide, offering a promising strategy to maintain glycemic control while reducing drug dosage. Although the primary aim of this study was to evaluate the adjunctive effect of electroacupuncture on semaglutide therapy, future studies including a sham electroacupuncture group will be valuable for further confirming the specificity of the observed effects.

## 5 Conclusion

**Figure 8:**
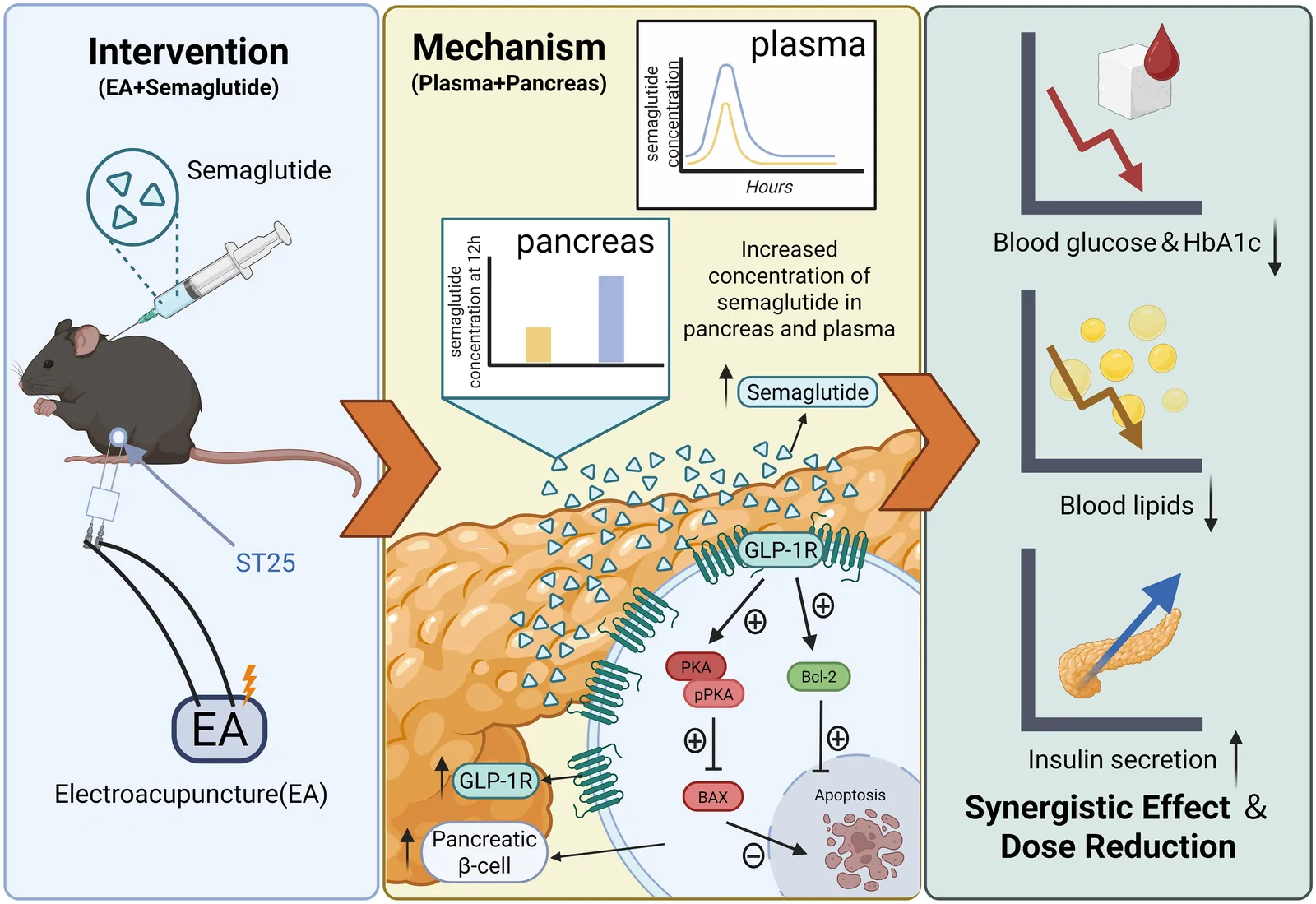
Synergistic effect of electroacupuncture and semaglutide on improving metabolic homeostasis and protecting pancreatic β-cells.

In summary, our findings provide the first evidence that EA combined with normal-dose or low-dose semaglutide exerts a synergistic effect, achieving glycemic control comparable to that of high-dose semaglutide. The combined intervention not only enhances the concentrations of semaglutide in plasma and pancreas but also protects pancreatic β-cells from apoptosis, likely through activation of the GLP-1R/PKA/Bax/Bcl-2 signaling pathway (**Figure 8**). EA is a promising adjunctive strategy that may enhance the therapeutic efficacy of semaglutide and provides preclinical evidence supporting future dose-sparing approaches in T2DM.

## Data Availability

The raw data supporting the conclusions of this article will be made available by the authors, without undue reservation.
