# Supplementary material for: Electroacupuncture enables semaglutide dose sparing in type 2 diabetes by boosting drug concentration and suppressing β-cell apoptosis
Source: Front Endocrinol (Lausanne). 2026 Aug 26;17:1880819. doi: 10.3389/fendo.2026.1880819 (PMC13553360; doi:10.3389/fendo.2026.1880819)
Supplement: Supplementary file 6 [file DataSheet6.zip › raw data/ethics/1.pdf]

# 南京中医药大学动物实验伦理审查申请表

申请号：202310A073

| 一、项目基本信息 | | | |
|---|---|---|---|
| 项目名称 | 基于肠道菌群- Treg/γδT细胞-IL-17免疫炎症信号通路的电针抗缺血性脑损伤机制研究 傅淑平 | | |
| 项目编号 | 012071001545 | | |
| 项目来源（请填写资助单位） | | | |
| 项目起止日期 | | | |
| 实验周期 | 60天 | | |
| 二、项目负责人信息 | | | |
| 姓名 | 傅淑平 | 部门/学院 | 南京中医药大学第二临床医学院 |
| 技术职称 | | 联系方式 | 18355138002 |
| 实验动物从业人员上岗证号 | | 电子邮箱 | 2724616107@qq.com |

| 三、动物实验操作者信息（参与活体动物实验操作的人员均需填写） | | | | |
|---|---|---|---|---|
| 姓名 | 单位（科室） | 技术职称 | 本实验中承担的操作 | 实验动物从业人员上岗证号 |
| 吴布凡 | 针灸推拿学院 | 硕士 | 造模、针灸 | 220212967 |
| 吴嘉悦 | 针灸推拿学院 | 硕士 | 造模、针灸 | 220234737 |
| | | | | |
| | | | | |

| 四、实验负责人（由项目负责人指定，或为负责人本人） | | | |
|---|---|---|---|
| 姓名 | 陈泳琳 | 电子邮件 | 1399320137@qq.com |
| 移动电话 | 15851485156 | 固定电话 | 15851485156 |

| 五、动物实验概况 | |
|---|---|
| 动物实验名称 | 电针联合司美格鲁肽治疗2型糖尿病的增效减药机制研究 |
| 本实验的目的和必要性 | 糖尿病是全球高发慢性病中患病人数增长最快的疾病，是导致心血管疾病、失明、肾衰竭以及截肢等重大疾病的主要病因之一。目前全球有5.4亿成人糖尿病患者，中国占据四分之一，是糖尿病第一大国。前期研究结果表明电针“天枢”可通过调节肠神经系统中氮能神经元的数量，有效改善2型糖尿病小鼠肠道黏膜损伤，恢复肠运动功能。据此本研究拟以2型糖尿病模型为载体，以链脲霉素诱导2型糖尿病，司美格鲁肽作为药物部分的治疗，进行电针联合司美格鲁肽治疗2型糖尿病的增效减药机制研究。本研究将小鼠分为模型组，正常使用剂量组，减量使用剂量组，针刺+正常使用剂量组，针刺+减量使用剂量组，超短波理疗仪组。每组15只，共90只。通过western blot和qPCR和免疫荧光等技术检测，探究电针联合司美格鲁肽治疗2型糖尿病是否可以增效减药，丰富“针灸治疗”的现代科学内涵，以期为针刺促进增效减药提供实验依据。 |
| 动物实验方法（按本中心要求整理成附件上传） | 58330dd4d0d7d29c&糖尿病小鼠dbdb鼠 (1).docx&.docx |
| 动物品系数量 | 1 |
| 品种/品系及选择依据 | db/db小鼠，依据参考文献选择 |
| 动物分组及各组数量 | 模型组，正常使用剂量组，减量使用剂量组，针刺+正常使用剂量组，针刺+减量使用剂量组，超短波理疗仪组。每组10只，共60只。 |
| 疾病动物模型 | 2型糖尿病小鼠模型 |
| 动物实验类别 | 常规 |
| 动物尸体处理 | 委托动物实验设施所属单位统一处理 |
| 涉及动物福利伦理的其他问题或对审查的特殊要求 | 无 |
| 六、动物实验详情（本部分仅显示一个品种/品系，使用多品种/品系时请查看增补表） | |

<table>
<tr><td>是否基因修饰动物</td><td colspan="4">基因修饰动物Leptin受体点突变，会引起小鼠患上2型糖尿病</td></tr>
<tr><td>动物信息</td><td colspan="4">江苏集萃药康 SPF 级 BKS-DB（dbdb）纯合小鼠 6-8周</td></tr>
<tr><td>动物用量</td><td>雄性</td><td>60只</td><td>雌性</td><td>0只</td></tr>
<tr><td>动物来源</td><td colspan="4">采购</td></tr>
<tr><td>动物实验设施</td><td colspan="4">屏障设施</td></tr>
<tr><td>饲养管理</td><td colspan="4">需高脂饲料喂养特殊饲养</td></tr>
<tr><td>实验终点</td><td colspan="4">固定时间点：在适应性喂养一周结束后，干预8周，随后取材</td></tr>
<tr><td>实验操作（本栏仅供选择操作项目，每项操作均请按提示以附件形式提交详细步骤）</td><td colspan="4">1、给药：超过一种实验操作：-皮下注射，针灸穴位，吸入麻醉; 2、采血：超过一种实验操作：-眼底静脉采血，尾静脉采血; 3、采集活体组织：无-; 4、手术：无; 5、为辅助实验操作而进行饮食和身体限制：禁食; 6、行为学项目：无-; 7、心理应激项目：无-; 8、其他操作：有：-针刺+正常使用剂量组，针刺+减量使用剂量组采用双极针进行针刺，“天枢”直刺 3-5 mm, 针刺入后不行针，接韩氏电针仪 2401，疏密波，频率 2/15 Hz, 电流强度 2mA,每次20min，每周6次，共8周。正常使用剂量组，减量使用剂量组，针刺+正常使用剂量组，针刺+减量使用剂量组皮下注射司美格鲁肽</td></tr>
<tr><td>预期本实验对动物的伤害及其处理预案</td><td colspan="4">糖尿病造模成功后，动物可能出现的临床症状包括：1. 频繁饮水和尿频：糖尿病小鼠由于高血糖，可能会出现频繁饮水和尿频的症状。2. 体重下降：由于糖尿病造成的代谢异常，小鼠可能会出现体重下降的情况。3. 疲劳和活动减少：糖尿病小鼠可能会出现疲劳和活动减少的症状。4. 毛发问题：糖尿病小鼠可能会出现毛发稀疏、毛色变化等毛发问题。5. 伤口愈合问题：糖尿病小鼠可能会出现伤口愈合缓慢或者愈合不良的情况。<br>在实验过程中，如果动物无法耐受实验，需要实施安乐死的仁慈终点指标可能包括：1. 体重下降：如果动物出现持续的体重下降超过20%，且不能通过调整饮食等方式改善，可能需要考虑实施安乐死。2. 严重的疼痛：如果动物出现严重的疼痛症状，导致虚弱无法进食，无法缓解，可能需要考虑实施安乐死。3. 严重的活动障碍：如果动物出现严重的活动障碍，无法正常行走或者活动，导致虚弱无法进食，可能需要考虑实施安乐死</td></tr>
<tr><td>预期实验操作中的意外情况及其处理预案</td><td colspan="4">无</td></tr>
<tr><td>安乐死方法</td><td colspan="4">颈椎脱臼</td></tr>
</table>
