# Supplementary material for: Electroacupuncture enables semaglutide dose sparing in type 2 diabetes by boosting drug concentration and suppressing β-cell apoptosis
Source: Front Endocrinol (Lausanne). 2026 Aug 26;17:1880819. doi: 10.3389/fendo.2026.1880819 (PMC13553360; doi:10.3389/fendo.2026.1880819)
Supplement: Supplementary file 6 [file DataSheet6.zip › raw data/ethics/2.pdf]

南京中医药大学动物实验伦理审查申请表

申请号：202509A061

一、项目基本信息			
项目名称	电针联合司美格鲁肽治疗2型糖尿病大鼠的增效减药机制研究		
项目编号	012071003943		
项目来源（请填写资助单位）			
项目起止日期			
实验周期	4天		
二、项目负责人信息			
姓名		部门/学院	
技术职称		联系方式	
实验动物从业人员上岗证号		电子邮箱	

三、动物实验操作者信息（参与活体动物实验操作的人员均需填写）

姓名	单位（科室）	技术职称	本实验中承担的操作	实验动物从业人员上岗证号
吴布凡	针灸推拿学院	硕士	给药	220212967
吴嘉悦	针灸推拿学院	硕士	饲养	220234737
杨心仪	针灸推拿学院	硕士	针灸	220236418

四、实验负责人（由项目负责人指定，或为负责人本人）			
姓名	杨心仪	电子邮件	1399320137@qq.com
移动电话	15851485156	固定电话	15851485156

五、动物实验概况	
动物实验名称	电针联合司美格鲁肽干预大鼠后组织内药物浓度和分布的研究
本实验的目的和必要性	糖尿病是全球高发慢性病中患病人数增长最快的疾病，是导致心血管疾病、失明、肾衰竭以及截肢等重大疾病的主要病因之一。目前全球有5.4亿成人糖尿病患者，中国占据四分之一，是糖尿病第一大国。前期研究结果表明电针天枢、后三里（相当于足三里），可引起血糖、血脂、血清胰岛素、抵抗素显著降低，有效改善胰岛素抵抗。据此本研究拟以空白SD大鼠作为，司美格鲁肽作为药物部分的治疗，进行电针联合司美格鲁肽治疗后组织内浓度的变化。本研究将大鼠分为药物组-4h，药物组-8h，药物组-12h，药物组-24h，药物+天枢组-4h，药物+天枢组-8h，药物+天枢组-12h，药物+天枢组-24h，药物+足三里组-4h，药物+足三里组-8h，药物+足三里组-12h，药物+足三里组-24h。共12组，每组5只，共60只。通过液质联用等技术检测，探究电针联合司美格鲁肽干预正常大鼠血药浓度的变化，丰富“针灸治疗”的现代科学内涵，以期为针刺促进增效减药提供实验依据。
动物实验方法（按本中心要求整理成附件上传）	3804c397c9c61fbb&普通大鼠组织内浓度.docx&.docx&WF_EAM
动物品系数量	1
品种/品系及选择依据	SD大鼠，依据参考文献选择，[1]程天,解伟伟,张玉倩,等.LC-MS/MS法测定大鼠血浆中曲拉西利的浓度及药动学研究[J].中国新药杂志,2025,34(10):1110-1115.
动物分组及各组数量	药物组-4h，药物组-8h，药物组-12h，药物组-24h，药物+天枢组-4h，药物+天枢组-8h，药物+天枢组-12h，药物+天枢组-24h，药物+足三里组-4h，药物+足三里组-8h，药物+足三里组-12h，药物+足三里组-24h。共12组，每组5只，共60只。
疾病动物模型	不涉及
动物实验类别	常规
动物尸体处理	委托动物实验设施所属单位统一处理
涉及动物福利伦理的其他问题或对审查的特殊要求	无

六、动物实验详情（本部分仅显示一个品种/品系，使用多品种/品系时请查看增补表）

是否基因修饰动物	不基因修饰动物		
动物信息	上海必凯生物 SD大鼠 （3-8周龄）		
动物用量	雄性	60只	雌性	0只
动物来源	采购		
动物实验设施	屏障设施		
饲养管理	常规饲养		
实验终点	固定时间点：		
实验操作（本栏仅供选择操作项目，每项操作均请按提示以附件形式提交详细步骤）	1、给药：皮下注射,针灸穴位及方法,其他注射途径-异氟烷吸入麻醉大鼠（5%诱导麻醉后，以2%维持麻醉）; 2、采血：眼底静脉采血-; 3、采集活体组织：无-; 4、手术：无; 5、为辅助实验操作而进行饮食和身体限制：禁食; 6、行为学项目：无-; 7、心理应激项目：无-; 8、其他操作：有：-电针治疗处理方法：采用双极针进行针刺，“天枢”或“足三里”直刺3-5 mm, 针刺入后不行针，接韩氏电针仪，疏密波，频率2/15 Hz, 电流强度2mA, 20min，。		
预期本实验对动物的伤害及其处理预案	无		
预期实验操作中的意外情况及其处理预案	1. 体重下降：如果动物出现持续的体重下降超过20%，且不能通过调整饮食等方式改善，可能需要考虑实施安乐死。		
安乐死方法	过量麻醉：5%异氟烷诱导并维持麻醉至呼吸停止死亡		
